# COMMD10 Is Essential for Neural Plate Development during Embryogenesis

**DOI:** 10.3390/jdb11010013

**Published:** 2023-03-16

**Authors:** Khanh P. Phan, Panayiotis Pelargos, Alla V. Tsytsykova, Erdyni N. Tsitsikov, Graham Wiley, Chuang Li, Melissa Bebak, Ian F. Dunn

**Affiliations:** 1 Department of Neurosurgery, University of Oklahoma Health Sciences Center, Oklahoma City, OK 73104, USA; khanh-phan@ouhsc.edu (K.P.P.); panayiotis-pelargos@ouhsc.edu (P.P.); alla-tsytsykova@ouhsc.edu (A.V.T.); erdyni-tsitsikov@ouhsc.edu (E.N.T.); 2 Clinical Genomics Center, Oklahoma Medical Research Foundation, Oklahoma City, OK 73104, USA; graham-wiley@omrf.org; 3 Genes & Human Disease Research Program, Oklahoma Medical Research Foundation, Oklahoma City, OK 73104, USA; chuang-li@omrf.org (C.L.); melissa-bebak@omrf.org (M.B.)

**Keywords:** COMMD10, Sox10, neural crest, embryonic development

## Abstract

The COMMD (copper metabolism MURR1 domain containing) family includes ten structurally conserved proteins (COMMD1 to COMMD10) in eukaryotic multicellular organisms that are involved in a diverse array of cellular and physiological processes, including endosomal trafficking, copper homeostasis, and cholesterol metabolism, among others. To understand the role of COMMD10 in embryonic development, we used *Commd10^Tg(Vav1-icre)A2Kio^*/J mice, where the *Vav1-cre* transgene is integrated into an intron of the *Commd10* gene, creating a functional knockout of *Commd10* in homozygous mice. Breeding heterozygous mice produced no COMMD10-deficient *(Commd10^Null^)* offspring, suggesting that COMMD10 is required for embryogenesis. Analysis of *Commd10^Null^* embryos demonstrated that they displayed stalled development by embryonic day 8.5 (E8.5). Transcriptome analysis revealed that numerous neural crest-specific gene markers had lower expression in mutant versus wild-type (WT) embryos. Specifically, *Commd10^Null^* embryos displayed significantly lower expression levels of a number of transcription factors, including a major regulator of the neural crest, *Sox10*. Moreover, several cytokines/growth factors involved in early embryonic neurogenesis were also lower in mutant embryos. On the other hand, *Commd10^Null^* embryos demonstrated higher expression of genes involved in tissue remodeling and regression processes. Taken together, our findings show that *Commd10^Null^* embryos die by day E8.5 due to COMMD10-dependent neural crest failure, revealing a new and critical role for COMMD10 in neural development.

## 1. Introduction

Endosomes are intracellular lipid bilayer organelles that regulate the trafficking of biological cargo between the plasma membrane and other subcellular compartments, including the *trans*-Golgi network and lysosomes. Following endocytosis, transmembrane proteins undergo sorting to be recycled back to the cell surface or sent for degradation in lysosomes. Cell surface recycling is essential for membrane receptor maintenance and is executed by two distinct protein complexes: Retromer and Retriever (reviewed in [1]). Each of these recycling complexes associates with other multi-protein structures, such as the Wiscott-Aldrich and Scar Homolog (WASH) complex and the COMMD/CCDC93/CCDC22 (CCC) complex [2,3]. Mutations in these multi-protein complexes are increasingly associated with human pathologies, including neurodegenerative and developmental disorders [4,5,6,7]. 

The COMMD (copper metabolism MURR1 domain)-containing subunit of the CCC complex includes several COMMD family proteins [1]. All members of this family share a unique C-terminal motif termed a COMM domain, which fosters homo- and hetero-dimerization of COMMD proteins and facilitates interactions with CCDC22 and CCDC93. On the other hand, the N-terminal region is unique in each COMMD protein, suggesting their diverse functions [8]. The first identified member of this family, COMMD1, was discovered to be mutated in Bedlington terriers with copper toxicosis [9]. Subsequently, COMMD1 was demonstrated to regulate the endosomal sorting of the copper transporter ATP7A [2]. COMMD1 also participates in the downregulation of nuclear factor kappa B (NF-κB)-dependent transcription [10,11].

The analysis of *Commd10* conditional knockout mice with targeted deficiency to myeloid cells and macrophages demonstrated its direct role in propagating phagolysosomal maturation and clearing of monocyte-driven inflammation [12] and infection [13]. However, *Commd10* is ubiquitously expressed, suggesting its role in other tissues [14,15]. Here, we examine the role of COMMD10 in the embryonic development of mice with a disrupted *Commd10* gene.

## 2. Materials and Methods

### 2.1. Mice

*Commd10^Het^* mice were bought from the Jackson Laboratory (B6.Cg-Commd10^Tg(Vav1-icre)A2Kio^/J, Stock # 008610) [16]. Wild-type (WT) and *Commd10^Null^* embryos were generated by interbreeding of *Commd10^Het^* littermates. Animals were housed and bred in a specific pathogen-free animal facility and fed a standard diet. All mouse breeding and procedures were carried out according to the laboratory animal protocol approved by the IACUC. Animal genotyping was based on the detection of the intact *Commd10* allele and *iCre* by real-time PCR using a DuPlex PCR approach with the following TaqMan assays:

Commd10-Fwd: CGGGTCTTCCCATCTCATTT

Commd10-Rev: TCAACTGGTTAGTCGGGATTG

Commd10 Probe: CAGACACACCCAGAGGCTCATTCATT

iCre-Fwd: TGGGCATTGCCTACAACA

iCre-Rev: ATCAGCATTCTCCCACCATC

iCre Probe: CGCATTGCCGAAATTGCCAGAATCA

### 2.2. Embryological Analysis

In order to harvest embryos at specified embryologic stages, timed pregnancies were set up by breeding *Commd10^Het^* mice. The embryos were considered 0.5 days post coitus (dpc) at noon on the day of detection of the vaginal plug. At embryonic days 8.5 (E8.5), E9.5, and E10.5, females were euthanized and embryos extracted. Embryonic genotyping was performed on genomic DNA purified from yolk sacs. Whole embryo images were obtained at total magnifications of 15× and 45× (combination of magnifications of 1.5× and 4.5× objective lens with 10× ocular lens) using an AmScope microscope with a MU1003 digital camera and AmScope software (AmScope).

### 2.3. Western Blot Analysis

Whole embryos were lysed in 1× Laemmli Sample Buffer (BIO-RAD, Hercules, CA, USA, 1610747) with 50 mM DTT, mixed with glass beads, and shaken in an Eppendorf shaker at 2000 RPM at 85 °C for 10 min. Samples were run on a 4–15% Mini-PROTEAN^®^ TGX™ Protein Gel (BIO-RAD, 4561083); transferred to nitrocellulose membranes, which were blocked with Blotting-Grade Blocker (BIO-RAD, 1706404); and probed with anti-COMMD10 (Fisher Scientific, Waltham, MA, USA, PIPA531868; RRID: AB_2549341), anti-COMMD1 (Fisher Scientific, PIPA598616; RRID: AB_2813229), or anti-Sp1 antibody (Sigma-Aldrich, St. Louis, MO, USA, 07-645). Goat anti-rabbit IgG, HPR-linked (Cell Signaling Technologies, Danvers, MA, USA, 7074) was used as a secondary antibody. Sp1 levels were measured as loading controls.

### 2.4. RNA Extraction

WT and *Commd10^Null^* embryos at days E8.5, E9.5, and E10.5 were extracted from yolk sacs and immediately placed in Invitrogen™ RNA*later*™ Stabilization Solution (Fisher Scientific, AM7023). They were kept at 4 °C for 24 h and transferred to −80 °C for long-term storage before RNA extraction. Total RNA was extracted using the RNeasy Plus Micro Kit (QIAGEN, Hilden, Germany, 74034) and QIAshredder (QIAGEN, 79656) according to the manufacturer’s instructions. 

### 2.5. RNA-seq and Differential Expression (DE) Analysis

Total RNA purified from WT and *Commd10^Null^* embryos at E8.5, E9.5, and E10.5 was subjected to full transcriptome sequencing. At least three biological repeats were carried out for each condition. 3′-end RNA libraries were made using the Lexogen QuantSeq 3′ mRNA-seq Library Prep Kit FWD for Illumina. Sequencing was performed from single-end 75bp on an Illumina NextSeq High Output.

Post-sequence reads were quality-filtered for length and contaminants and were trimmed for Illumina adapters using BBDuk [17]. The resulting reads were pseudo-aligned to coding regions of the mouse reference genome (mm10) using STAR [18]. Gene annotation was performed via the R package biomaRt [19]. Differential expression was calculated using the Wald test implemented in the R package DESeq2 [20]. Significantly differentially expressed genes were defined as those that had both an absolute log2Fold change ≥ 1 and a false discovery rate (FDR) adjusted *p*-value ≤ 0.05 for each comparison independently.

### 2.6. Quantitative PCR (RT-qPCR)

Whole embryo total RNA was used to measure gene mRNA levels by real-time qPCR. Reverse transcription and cDNA amplification were performed in one tube using qScript™ XLT One-Step RT-qPCR ToughMix^®^, Low ROX™ (VWR Quanta Biosciences™, Beverly, MA, USA, 95134) on an Applied Biosystems 7500 Fast Real-Time PCR System (Fisher Scientific). Sample reactions were run in 3–6 replicates. Each mRNA analysis was run in a DuPlex PCR reaction with *Gapdh* as an internal control. Standard curves for each gene were run to verify the linear range of amplification. Input RNA was kept under 200 ng per reaction to stay within the linear range for *Gapdh* levels. 

All data were analyzed in Microsoft Excel with the built-in analysis methods. TaqMan assays used for RT-qPCR are as follows (m–mouse assays):

mGapdh-Fwd: CCTGTTGCTGTAGCCGTATT

mGapdh-Rev: AACAGCAACTCCCACTCTTC

mGapdh Probe: TTGTCATTGAGAGCAATGCCAGCC

mSox10-Fwd: GCTATTCAGGCTCACTACAAGA

mSox10-Rev: GGACTGCAGCTCTGTCTTT

mSox10 Probe: ATGTCAGATGGGAACCCAGAGCAC

## 3. Results and Discussion

**Figure 1:**
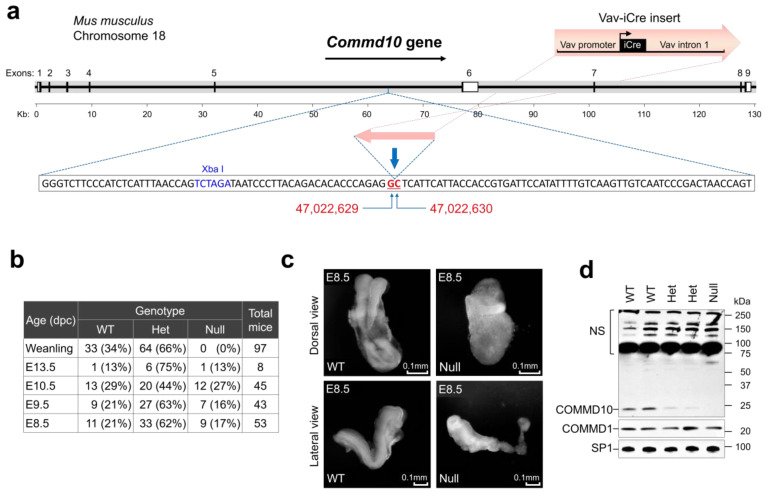
COMMD10 deficiency results in embryonic lethality. (**a**) Schematic drawing (up-to-scale) of the *Commd10* gene on mouse chromosome 18 shown as a thick grey line. Its direction of transcription is indicated by the black arrow above. Coding exons are represented as thin black boxes. Noncoding 5′- and 3′-untranslated regions are shown as open boxes. The Vav-iCre cassette sketch is shown above the track. The sequence around the Vav-iCre cassette insertion site is shown below the gene scheme in an inset window. Flanking the cassette, GC nucleotides are marked by red bold underlined font and indicated by blue arrows. Their exact positions in the genome are designated by numbers from Reference GRCm39 C57BL/6J below the sequence window. (**b**) Genotyping analysis of offspring of heterozygous *Commd10^Het^* mice (Het) mating. *Commd10^Null^* (Null) mice had never been born but embryo genotypes show the expected Mendelian distribution. (dpc): days post-coitus. (**c**) Morphological analysis of WT and *Commd10^Null^* (Null) embryos at E8.5 in dorsal (top panels) and lateral (bottom panels) views. (**d**) Western blot analysis of whole embryo lysates and anti-COMMD10 or anti-COMMD1 antibodies, as indicated. Anti-SP1 antibody was used as the loading control. NS: non-specific bands.

To examine the role of COMMD10 in embryonic development, we used B6.Cg-*Commd10^Tg(Vav1-icre)A2Kio^*/J mice (Jackson Laboratory; stock #008610). In these mice, the *Vav1-iCre* transgene is integrated into the intron between exons 5 and 6 of the *Commd10* gene on chromosome 18 (Figure 1a) [16]. The insertion resulted in a functional knockout of *Commd10* in homozygous (*Commd10^Null^*) mice [21]. Crossbreeding of *Commd10* heterozygous (*Commd10^Het^*) littermates produced no *Commd10^Null^* newborn mice, while WT and heterozygous genotypes were born at the expected Mendelian ratio (Figure 1b). These results are consistent with those of a viability primary screen phenotypic assay performed on another *Commd10* mutant mouse strain (*Commd10^tm1a(EUCOMM)Wtsi^*) from the EUCOMM consortium (strain #EPD065) at https://www.mousephenotype.org/data/genes/MGI:1916706 (accessed on 17 July 2022). However, the phenotype of these mice has not been reported in the literature. Thus, the essential role of COMMD10 in embryonic development was confirmed by using two different mouse strains with deficient COMMD10 expression.

E8.5 *Commd10^Null^* embryos were visually abnormal and displayed abnormal neural plate morphology and growth retardation, but still remained comparable in size and yielded a comparable amount of RNA for analysis (Figure 1c). E9.5 and E10.5 mutant embryos showed progressive degradation and signs of tissue resorption (Figure S1a). Western blot analysis of E8.5 embryo lysates demonstrated lower levels of COMMD10 protein in *Commd10^Het^* embryos and its complete absence in *Commd10^Null^* embryos compared with WT embryos (Figure 1d). 

**Figure 2:**
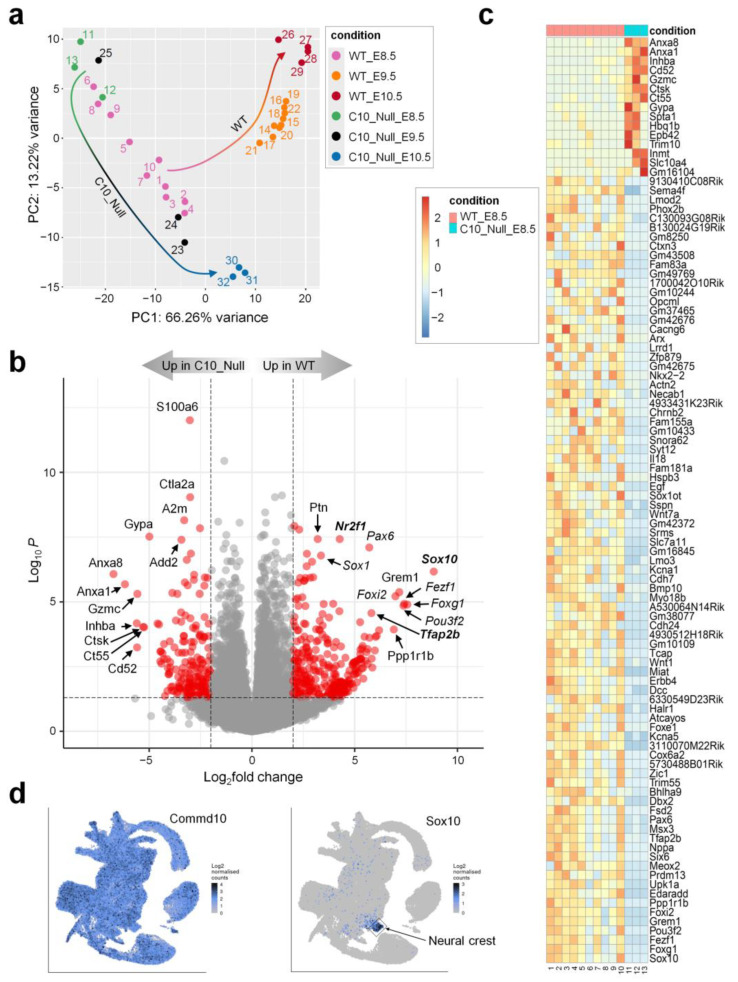
*Commd10^Null^* embryos fail to develop beyond E8.5 due to impaired neural plate and neural crest development. (**a**) PCA plot of RNA-seq analysis in WT and *Commd10^Null^* (C10_Null) embryos at E8.5, E9.5, and E10.5. Sample clusters are shown in different colors. Colored arrows show direction of cluster shifts through E8.5 to E10.5 developmental timeframe for both genotypes. Changing arrow colors correlate with the corresponding sample cluster in a timeframe. (**b**) Volcano plot of RNA-seq analysis visualizing significant DEGs in WT vs. *Commd10^Null^* (C10_Null) E8.5 embryos: magnitude of change (x-axis) vs. statistically significant *p*-values (y-axis). Points that have a fold change less than 2 (log_2_ = 1) are shown in grey. Genes that are transcription factors are marked in italic font. Genes that are expressed in neural crest more highly than in any other cell type are shown in Bold font. (**c**) Heatmap of mRNA expression levels for top 100 significant DEGs in WT vs. *Commd10^Null^* E8.5 embryos by RNA-seq. (**d**) Distribution of *Commd10* and *Sox10* mRNA expression in WT embryos during early embryogenesis in a single-cell molecular map [22]. Presented plots were generated on a single-cell molecular map of mouse gastrulation and early organogenesis at https://marionilab.cruk.cam.ac.uk/MouseGastrulation2018/ (accessed on 2 September 2022). The full legend annotating cell clusters by different colors and the schematic map are shown in Figure S1c.

To examine the root cause of the developmental failure of *Commd10^Null^* embryos, we carried out comparative transcriptome analyses of mutant and WT embryos (Figure S1b). Figure 2a shows the gene expression principal component analysis (PCA) plot. The cluster of WT samples on E8.5 appears stretched compared with other clusters, indicating some variability among WT samples on that day. The rest of the clusters are tight without any overlap. Importantly, the direction of embryonic development from E8.5 through E10.5 is reflected in the WT cluster distribution on the PCA plot (WT arrow). Interestingly, *Commd10^Null^* E8.5 and E9.5 clusters are located on opposite sides of the WT E8.5 samples. Importantly, both of these clusters are far from each other and from E10.5 samples (Figure 2a, C10_Null arrow). This segregation pattern suggests that the divergence point between WT and *Commd10^Null^* embryos took place not long before day E8.5. Thus, the *Commd10^Null^* E8.5 transcriptome represents an inflection point in embryogenesis from development to tissue resorption. 

Figure 2b shows a volcano plot visualizing differentially expressed genes (DEGs) in WT vs. *Commd10^Null^* embryos at E8.5 and displaying wide areas of scattered genes on both sides of the y-axis. We sorted all significant DEGs by the absolute value of log2FoldChange and chose the top 100 DEGs to plot on a heatmap (Figure 2c). Among these top 100 DEGs, only 15 were upregulated in *Commd10^Null^* embryos, and the 85 remaining genes were downregulated in contrast to those in WT embryos. Interestingly, the 85 DEGs that are downregulated in mutant embryos include 20 transcription factors, at least 11 cytokines/growth factors/cell surface receptors, and 30 genes with unknown function. The rest of these DEGs encode structural proteins, modifying enzymes, and proteins involved in ion channel function, cell adhesion, and other metabolic cellular processes. 

**Figure 3:**
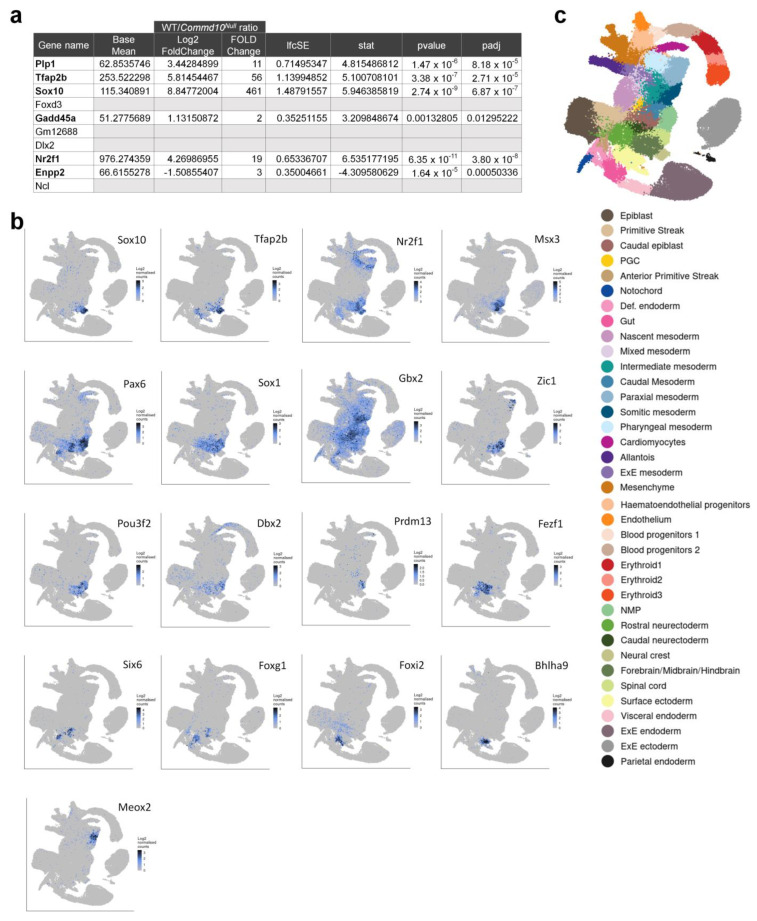
Six of the top ten neural crest-specific markers are differentially expressed in WT versus *Commd10^Null^* embryos. (**a**) Table listing the top ten neural crest-specific markers, genes that are expressed in the neural crest more highly than in any other cell type. Six genes with differential expression in WT and *Commd10^Null^* embryos are shown in bold font. (**b**) Tissue distribution of mRNA expression of different transcription factors in WT embryos during early embryogenesis in the molecular map of whole dataset, as described in (**c**). (**c**) Legend for (**b**) annotating cell clusters by different colors, and a single-cell molecular map of mouse gastrulation and early organogenesis [22] up to day E8.5 of embryogenesis. All presented plots were generated on a single-cell molecular map of mouse gastrulation and early organogenesis at https://marionilab.cruk.cam.ac.uk/MouseGastrulation2018/ (accessed on 2 September 2022).

To find the specific embryonic lineage where each of these DEGs is expressed, we searched a single-cell molecular map of mouse gastrulation and early organogenesis at https://marionilab.cruk.cam.ac.uk/MouseGastrulation2018/ (accessed on 2 September 2022) [22]. This interactive atlas demonstrates specific mRNA expression profiles during mouse embryonic development between E6.5 and E8.5. As shown in Figure 2d, *Commd10* is broadly expressed in all lineages during embryogenesis. The top most significantly (461-fold) downregulated gene in *Commd10^Null^* embryos at E8.5 is Sox10, a transcription factor with a central role in neural crest development and maturation of glia [23]. We have also validated Sox10 mRNA expression in WT and *Commd10^Null^* embryos at E8.5, E9.5, and E10.5 by RT-qPCR and found the highest Sox10 expression and the most drastic difference between the two genotypes at E8.5 (Figure S1d). In normal developing mouse embryos, Sox10 expression emerges after E8.0 almost exclusively in the neural crest (Figure 2d). The table in Figure 3a lists the top ten neural crest-specific markers according to the interactive atlas. Interestingly, six of those markers were differentially expressed in WT versus *Commd10^Null^* embryos, suggesting that there is a defect in neural crest development in *Commd10^Null^* embryos (Figure 2b–d and Figure 3b). Moreover, a list of significant DEGs, which define the trajectory of neurogenesis, includes numerous transcription factors critical for neural plate development, starting from rostral neuroectoderm at E6.5 and subsequent development of caudal neuroectoderm, spinal cord, forebrain/midbrain/hindbrain, and neural crest by E8.5. 

Besides Sox10, *Commd10^Null^* embryos exhibit significantly lower expression of transcription factors Tfap2b [24,25], Nr2f1 [26], Msx3 [27], Dbx2 with Pax6 [28,29], Sox1 [30], Gbx2 [31], Zic1 [32], Pou3f2 [33,34,35], Prdm13 [36], Fezf1 [37], Six6 [38,39], Foxg1 [40], and Foxi2 [41] (Figure 3). They all participate in the early stages of central nervous system development. Also significantly downregulated in *Commd10^Null^* embryos are genes encoding cytokines/growth factors involved in early embryonic neurogenesis, such as Ptn [42,43], Mdk [43,44], and Grem1 [45] (Figure 2b). In addition, transcription factors such as Meox2 [46], expressed in paraxial and somatic mesoderm, and Bhlha9 [47,48], expressed in surface ectoderm, are important for the expression of genes involved in signaling pathways essential for the formation and morphogenesis of somites and limbs in developing embryos (Figure 3). Taken together, these data are in agreement with the observation that WT embryos at E8.5 undergo continuous embryogenesis by means of cell proliferation, migration, and differentiation, particularly in the process of primary neurulation. This highly orchestrated process is defined by the expression of a number of transcription and growth factors that are coordinated in place and time. Significantly lower levels of these molecules in *Commd10^Null^* embryos may result in the termination of embryonic development. 

**Figure 4:**
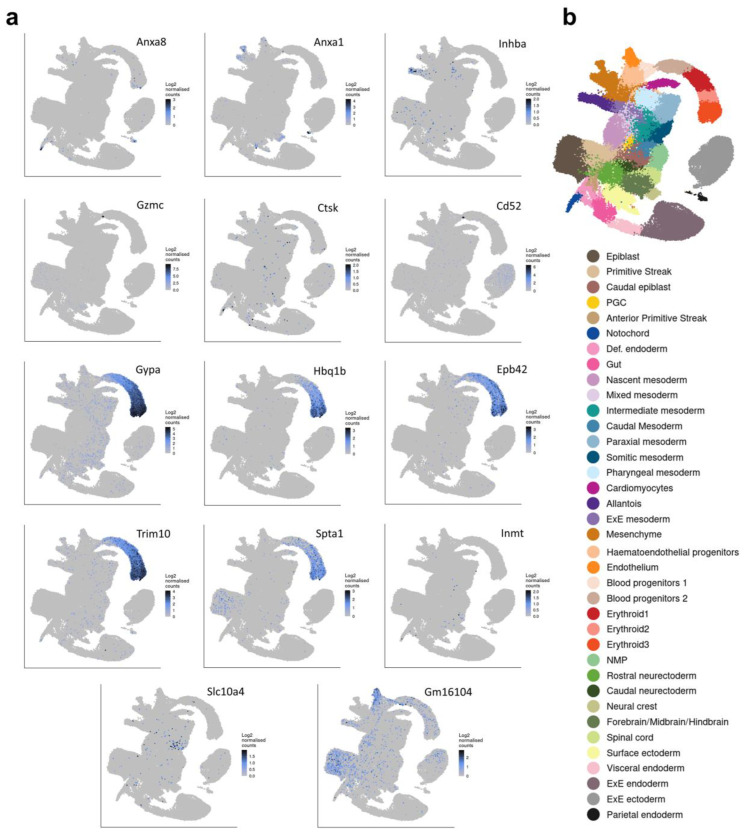
Tissue distribution of 14 genes significantly upregulated in *Commd10^Null^* embryos on E8.5. (**a**) Single-cell molecular maps of mRNA expression in WT embryos for the top 14 genes significantly upregulated in *Commd10^Null^* embryos on E8.5. (**b**) Legend for (**a**) annotating cell clusters by different colors, and a single-cell molecular map of mouse gastrulation and early organogenesis [22] up to day E8.5 of embryogenesis. All presented plots were generated on a single-cell molecular map of mouse gastrulation and early organogenesis. at https://marionilab.cruk.cam.ac.uk/MouseGastrulation2018/ (accessed on 2 September 2022) website.

**Figure 5:**
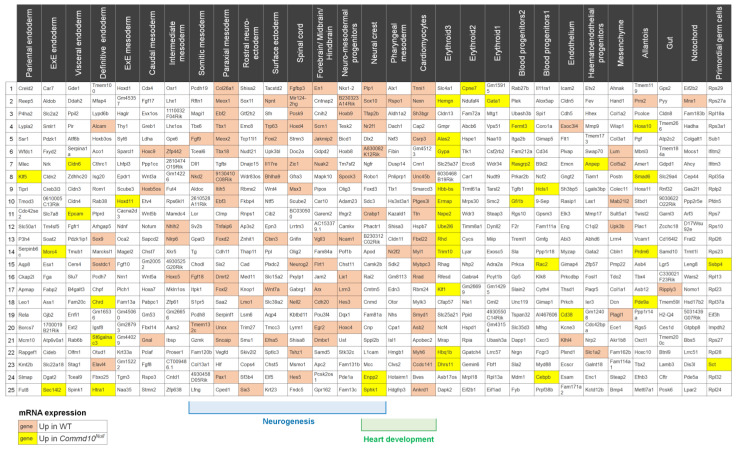
Tissue distribution of differentially expressed genes in *Commd10^Null^* embryos on E8.5. The top row of table lists 29 cell lineages/tissues present in normal mouse embryos at the E8.5 stage of embryogenesis. The columns list the top 25 lineage-specific gene markers for each tissue. All lists were found on a single-cell molecular map of mouse gastrulation and early organogenesis. at https://marionilab.cruk.cam.ac.uk/MouseGastrulation2018/ (accessed on 22 October 2022) website. Genes that are significantly expressed at lower levels in *Commd10^Null^* embryos when compared with WT are shaded in red. Genes with higher expression in *Commd10^Null^* are shaded in yellow. Blue and green brackets below the table mark cell lineages/tissues involved in neurogenesis and heart development, respectively.

On the other hand, there are no transcription factors or cytokine/growth factors among the top 15 DEGs upregulated in *Commd10^Null^* embryos at E8.5 as compared with their WT littermates (Figure 2c). While some of these genes, such as Anxa8 and Anxa1 [49], are modestly expressed in notochord, caudal neuroectoderm, and neural crest of the WT embryos, most are not expressed in developing neural tissue (Figure 4 and Figure 5). Instead, the majority of those genes are expressed in blood progenitors and erythroid tissue in particular (Gypa, Hbq1b, Epb42, Trim10 [50,51], Spta1). Interestingly, some of the upregulated DEGs in *Commd10^Null^* embryos may be involved in tissue remodeling and regression. Granzyme C (Gzmc) is increased 48-fold in *Commd10^Null^* embryos compared with WT, while Inhibin beta A chain (Inhba), a member of the inhibins/activins network of proteins, is increased 49-fold. Thus, embryonic cell death leading to tissue regression in E8.5 *Commd10^Null^* embryos may be caused by two main events. The first event is a failure of the neural plate and neural crest processes due to a substantial deficiency of transcription factor Sox10, together with lower expression of other transcription factors and cytokines/growth factors involved in early embryonic neurogenesis. The second event is based on the increased expression of proteins with potential embryo resorption abilities.

To verify our conclusions further, we examined the expression of statistically significant DEGs with the top 25 gene markers representing each embryonic cell type present in the mouse embryo at E8.5 (Figure 5). A single-cell molecular map of mouse gastrulation and early organogenesis [22] lists 29 different cell/tissue types for the E8.5 mouse embryo. The gene analysis revealed that the majority of genes with low expression in *Commd10^Null^* embryos are found in cells involved in early neural and heart development (Figure 5). Since recent studies demonstrated that neural crest cells develop into cardiomyocytes and contribute to heart development [52,53], gene expression deficiency in cardiomyocytes may be due to failed neural crest differentiation and/or cell migration.

We also performed gene functional enrichment analysis for the top 15–20 upregulated or downregulated DEGs using ToppGene Suite (https://toppgene.cchmc.org (accessed on 16 February 2023) [54]. We analyzed the top 20 genes downregulated in *Commd10^Null^* embryos and came up with a “GO: Biological Process” list of positive regulation of RNA biosynthetic process, epithelium development, animal organ morphogenesis, and brain and head development. We also analyzed the top 15 genes upregulated in *Commd10^Null^* embryos and selected the two top biological processes with the highest number of genes from the list: hemopoiesis and immune system development (Supplementary Tables).

Mice deficient in other members of the COMMD family, COMMD1 or COMMD9, were shown to be embryonically lethal. *Commd1^−/−^* embryos died between E9.5 and E10.5 due to defects in placenta vascularization [55]. Using genome-wide gene expression microarray analysis of embryonic RNA, the authors identified transcriptional upregulation of hypoxia-inducible factor 1 (HIF1) target genes in *Commd1^−/−^* embryos compared with their WT counterparts. Moreover, they demonstrated that COMMD1 may inhibit HIF1A stability and HIF1 activation by the physical association between the two proteins. Despite similarities in the timing of embryonic development failure between *Commd1^−/−^* and *Commd10^Null^* embryos, there were no similarities in gene expression patterns in the present study. Only *Pfkp*, one of eighteen hypoxia-associated DEGs upregulated in *Commd1^−/−^* versus WT embryos, was slightly upregulated in *Commd10^Null^* E8.5 embryos. Thus, the failure of *Commd10^Null^* embryos to thrive appears to have different underlying reasons compared to *Commd1*^−/−^ embryos. 

In contrast to *Commd10^Null^* embryos, *Commd9*^−/−^ embryos die by E13.5 [56]. The authors found low levels of Hey1, Hey2, and Hes1 mRNA in the hearts of *Commd9^−/−^* embryos and concluded that the embryonic lethality of these mice was due to complex cardiovascular changes with signs of Notch deficiency. There were no differences in the mRNA expression of Notch or the genes listed above in *Commd10^Null^* embryos compared with WT. Taken together, these data indicate that COMMD1-, COMMD9-, and COMMD10-deficient mice display different underlying reasons for failed embryonic development and suggest that COMMD proteins play different critical roles during embryogenesis.

No direct connection between COMMD10 and Sox10 has been described in the scientific literature. We can only speculate as to how the absence of COMMD10 may lead to lower expression of Sox10 and, sequentially, other genes during embryogenesis. During normal embryogenesis, Sox10 mRNA appears in late gastrulating embryos (mouse E7.5) in the neural crest-forming region, and its gene expression depends on Wnt signaling [57,58]. Sox10 protein was also found to directly interact with β-catenin [59], which is activated in the canonical Wnt signaling pathway (reviewed in [60]). Wnt protein ligands bind to Frizzled family receptors (cell surface Fzd proteins and co-receptor Lrp5/6). *Commd10^Null^* embryos show significantly lower expression of Fzd3 and Fzd9 suggesting lower Wnt signaling potency. In addition, several Wnt ligands themselves were also dysregulated. There were higher levels of Wnt3 and Wnt9b while there were significantly lower levels of Wnt1, Wnt7a, and Wnt8b, suggesting dysregulation of Wnt signaling pathways in *Commd10^Null^* embryos. Wnt1-deficient mice exhibit a range of phenotypes, from early embryonic lethality to survival with severe ataxia [61]. Wnt7a signaling also controls multiple steps of neurogenesis [62]. It is plausible that by being part of the endosomal trafficking process inside the cell, COMMD10 may be involved in Wnt signaling regulation through as yet unknown mechanisms of Fzd receptor recycling or Wnt ligand secretion.

## 4. Limitations of the Study

The results described here characterize the timing of embryonic lethality of *Commd10^Null^* mice and also begin to demonstrate that neural plate developmental delay is the most likely cause of *Commd10^Null^* failed embryogenesis. The differential gene expression profile of *Commd10^Null^* as compared to normally developing WT embryos after E8.5 does not necessarily imply direct associations with COMMD10 deficiency. They rather verify the timing of embryonic failure by E8.5. Broader approaches and detailed analyses of earlier embryos are needed to pinpoint the exact role of COMMD10 in mouse embryogenesis, which are subjects of continued study and outside the scope of the present study.

## 5. Conclusions

Our study demonstrated that COMMD10 deficiency leads to embryonic lethality by day E8.5, most likely due to impaired neural plate and neural crest development processes resulting from the decreased expression of transcription factor Sox10 and several other genes. The molecular mechanism by which COMMD10 upregulates *Sox10* expression remains unknown and merits further investigation.

## Data Availability

Most data generated or analyzed during this study are included in this published article and its Supplementary Materials. Unprocessed RNA-seq raw data files and processed data files have been deposited on NCBI Gene Expression Omnibus (https://www.ncbi.nlm.nih.gov/geo/query/acc.cgi?acc=GSE216492 (accessed on 13 March 2023). Further information and requests for materials should be directed to and will be fulfilled by the lead contact, Ian F. Dunn (ian-dunn@ouhsc.edu).

## Supplementary Materials

The following supporting information can be downloaded at: https://www.mdpi.com/article/10.3390/jdb11010013/s1, Figure S1: Complementary information for main figures; Supplementary Tables: GO:Biological Process of the top 15 genes upregulated in *Commd10^Null^* embryos and the GO:Biological Process of the top 20 genes downregulated in *Commd10^Null^* embryos; and a document with Supplementary figure titles and legends.
