# Supplementary material for: COMMD10 Is Essential for Neural Plate Development during Embryogenesis
Source: J Dev Biol. 2023 Mar 16;11(1):13. doi: 10.3390/jdb11010013 (PMC10051640; doi:10.3390/jdb11010013)
Supplement: Supplementary file 1 [file jdb-11-00013-s001.zip › Supplementary figure titles and legends.pdf]

Supplementary figure/tables titles and legends

Figure S1. Complementary information for main figures.

(**a**) Morphological analysis of wild-type (WT) and *Commd10*^{Null} (Null) embryos at E9.5. White scale bar is 0.1 mm. The *Commd10*^{Null} embryo is shown at higher magnification to visualize the degrading structure of the failed embryo.

(**b**) List of RNA samples from WT and *Commd10*^{Null} mouse embryos used in RNA-seq analysis presented in Figure 2.

(**c**) Full legend for Figure 2d annotating cell clusters by different colors, and a schematic single-cell molecular map of mouse gastrulation and early organogenesis up to day E8.5 of embryogenesis [22]. Presented legend picture is from a single-cell molecular map of mouse gastrulation and early organogenesis (cam.ac.uk) website.

(**d**) mRNA expression analysis of Sox10 in WT and *Commd10*^{Null} embryos at E8.5, E9.5, and E10.5. Total RNA was purified from whole mouse embryos and mRNA was amplified by RT-qPCR using GAPDH as internal control. Data are presented as relative quantity (RQ) Mean ± SE.

Supplementary Tables. GO:Biological Process of the top 15 genes upregulated in *Commd10*^{Null} embryos and the **GO:Biological Process of the top 20 genes downregulated in *Commd10*^{Null} embryos.** Gene functional enrichment analysis for the top 15-20 upregulated or downregulated DEGs was performed using ToppGene Suite (https://toppgene.cchmc.org) [54]. Both tables contain Hyperlinks to webpages with more detailed information about the analyses in both spreadsheet and graphical forms online.
