# Supplementary figures and images for: COMMD10 Is Essential for Neural Plate Development during Embryogenesis
Source: J Dev Biol. 2023 Mar 16;11(1):13. doi: 10.3390/jdb11010013 (PMC10051640; doi:10.3390/jdb11010013)

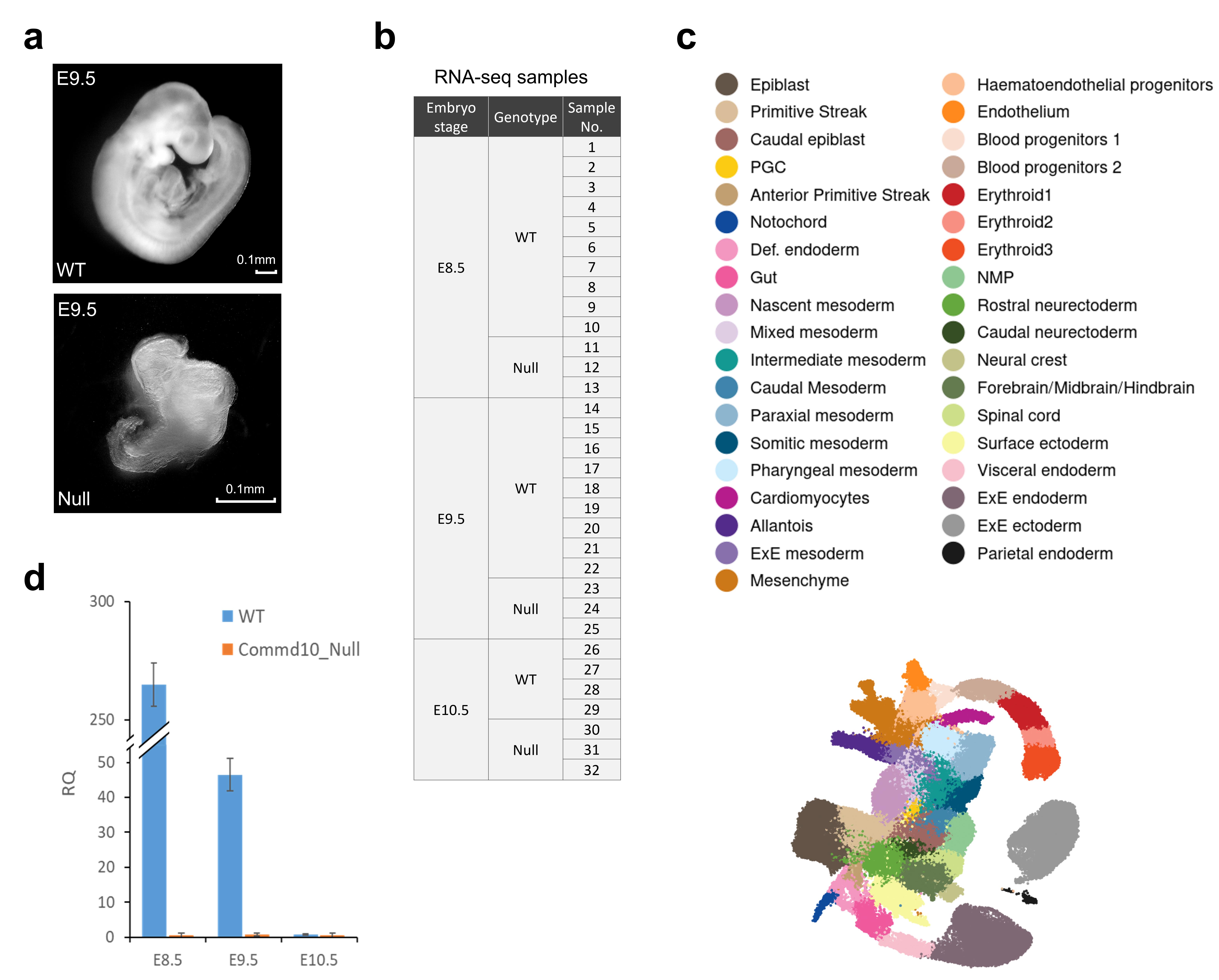

Supplement: Supplementary file 1 [file jdb-11-00013-s001.zip › Phan et al_FIGURE S1.rev1.jpg]
